# Withholding and withdrawing life-sustaining therapy in a Moroccan Emergency Department: An observational study

**DOI:** 10.1186/1471-227X-11-12

**Published:** 2011-08-12

**Authors:** Nada Damghi, Jihane Belayachi, Badria Aggoug, Tarek Dendane, Khalid Abidi, Naoufel Madani, Aicha Zekraoui, Abdellatif Benchekroun Belabes, Amine Ali Zeggwagh, Redouane Abouqal

**Affiliations:** 1 Medical Emergency Department, Ibn Sina University Hospital, 10000, Rabat, Morocco; 2 Medical Intensive Care Unit, Ibn Sina University Hospital, 10000, Rabat, Morocco; 3 surgical Emergency Department, Ibn Sina University Hospital, 10000, Rabat, Morocco; 4 Laboratory of Biostatistics, Clincial and Epidemiological Research, Faculté de Médecine et Pharmacie - Université Mohamed V, 10000, Rabat, Morocco

**Keywords:** Emergency, life-sustaining treatment, withdrawal, withholding

## Abstract

**Background:**

Withdrawing and withholding life-support therapy (WH/WD) are undeniably integrated parts of medical activity. However, Emergency Department (ED) might not be the most appropriate place to give end-of life (EOL) care; the legal aspects and practices of the EOL care in emergency rooms are rarely mentioned in the medical literature and should be studied. The aims of this study were to assess frequency of situations where life-support therapies were withheld or withdrawn and modalities for implement of these decisions.

**Method:**

A survey of patients who died in a Moroccan ED was performed. Confounding variables examined were: Age, gender, chronic underlying diseases, acute medical disorders, APACHE II score, Charlson Comorbidities Index, and Length of stay. If a decision of WH/WD was taken, additional data were collected: Type of decision; reasons supporting the decision, modalities of WH/WD, moment, time from ED admission to decision, and time from processing to withhold or withdrawal life-sustaining treatment to death. Individuals who initiated (single emergency physician, medical staff), and were involved in the decision (nursing staff, patients, and families), and documentation of the decision in the medical record.

**Results:**

177 patients who died in ED between November 2009 and March 2010 were included. Withholding and withdrawing life-sustaining treatment was applied to 30.5% of all patients who died. Therapies were withheld in 24.2% and were withdrawn in 6.2%. The most reasons for making these decisions were; absence of improvement following a period of active treatment (61.1%), and expected irreversibility of acute disorder in the first 24 h (42.6%). The most common modalities withheld or withdrawn life-support therapy were mechanical ventilation (17%), vasopressor and inotrops infusion (15.8%). Factors associated with WH/WD decisions were older age (OR = 1.1; 95%IC = 1.01-1.07; *P *= 0.001), neurological acute medical disorders (OR = 4.1; 95%IC = 1.48-11.68; P = 0.007), malignancy (OR = 7.7; 95%IC = 1.38-8.54; *P *= 0.002) and cardiovascular (OR = 3.4;95%IC = 2.06-28.5;*P *= 0.008) chronic underlying diseases.

**Conclusion:**

Life-sustaining treatment were frequently withheld or withdrawn from elderly patients with underlying chronic cardiovascular disease or metastatic cancer or patients with acute neurological medical disorders in a Moroccan ED. Religious beliefs and the lack of guidelines and official Moroccan laws could explain the ethical limitations of the decision-making process recorded in this study.

## Background

The withholding and withdrawal of life-sustaining treatment (WH/WD) refer to the process by which medical interventions are not given or are removed from patients with the expectation that they will die as a result. These decisions are, for patient's physicians and relatives, difficult to take and depend on ethical issues related to legal, cultural, moral, and religious values [1-3]. Emergency medicine developed as a medical specialty to care for patients with acute illness or injury who require immediate intervention, and who would then be referred for definitive care [4]. As emergency visits by older adults with serious and complex illness continue to rise [5,6], emergency providers are increasingly caring for patients with exacerbations of chronic, advanced illness [4]. However, many patients die each year in ED and terminal care decisions are difficult to implement in the ED owing to the absence of an ongoing long-term relationship with the patient and lack of time [7-9]. Interactions between end-of-life models and emergency care have been explored by Chan [10], and even though the emergency department might not be the most appropriate place to give end-of life care.

Decisions to limit life support have been widely studied in critical care medicine [11-22]. Few data are available concerning this type of decision in the emergency departments (ED) [4,9,23,24]. However, to our knowledge, there are no studies concerning WH/WD life-sustaining therapy in ED from Arabic countries where religious and ethical values, medical resources are different from those in Western countries [25-27]. There are no guidelines in Morocco, where social traditions are rather conservative. Moreover, relations among family members are close, and religious issues often play a vital role in decision-making by families and physicians [27]. We undertook an observational study of practices in WH/WD in a Moroccan ED to assess the frequency of such practices, the therapies withheld or withdrawn, and the processes leading to these decisions.

## Methods

### Study design and setting

This was an observational study conducted in the Emergency Department (ED) of Rabat University Hospital, from November 2009 to March 2010. Ibn Sina university hospital in Rabat is the referral for habitants in Western-North Morocco, it is a 1028 bed tertiary - stage hospital that opened in 1955. The bed occupancy rate is of 76% to 85%. The hospital comprises 24 departments (12 surgical, 9 medicals, and 3 intensive care units), and admits adult patients. Gynecology-obstetric and pediatric patients are treated in other structures. The mean of ED visits (including consultation and admission) per day is 176. The ED comprises two units (medical and surgical). The medical staff is constituted by 4 senior doctors (greater than 2 years experience in the unit) and 5 juniors (emergency physicians, and resident juniors with less than 2 years experience in the emergency unit). All staff members who belonged to the ED were not aware of the progress of the study.

### Definitions

Withdrawal was defined as discontinuation of treatments that had previously been implemented, and withholding was defined as a predetermined decision not to implement therapies that would otherwise be deemed necessary: endotracheal intubation, mechanical ventilation, intravenous (IV) fluid expansion, massive transfusion (more than three red cell packs), vasopressor infusion, cardiopulmonary resuscitation, renal replacement therapy [28].

### Data collection

We surveyed all adult patients who died on stretchers after their admission to the ED. Patients with brain death were excluded and those who died during transit to the ED.

Data were collected by a single senior member who was never involved in the decision of withholding and withdrawal of life-sustaining treatment. He interviewed every day the doctor (who documented specifically his action) about all patient who died in emergency department in the last 24 hours.

Collected data, from each patient who died, included age and gender, chronic underlying diseases, acute medical disorders, the severity of illness at admission using the Acute Physiology and Chronic Health Evaluation II (APACHE II) score [29], the prior health condition status using Charlson Comorbidities Index (CCI)[30]. The chronic underlying diseases considered were: malignancy (defined as current malignancy with metastasis or with failure of curative treatment), Heart failure (defined as New York Heart Association class 4), chronic respiratory disease (defined as chronic restrictive or obstructive pulmonary disease), and Liver disease. Length of stay in ED was also recorded.

If a decision to limit life support was taken, additional data were collected: The type of decision whether it was withdrawal or withholding life-sustaining treatment. The reasons supporting such a decision were noted using pre-specified items: Principal acute presenting medical disorder, expected irreversibility of acute disorder in the first 24 h, age, previous functional limitation, underlying chronic disease; absence of improvement following a period of active treatment, underlying disease expected to be fatal in the following 6 months, recovery but expected quality of life unacceptably poor, level of care considered to be maximal (more aggressive therapy would be unreasonable), and high cost of care. Life-sustaining treatment modalities withheld or withdrawn were noted as: mechanical ventilation, endotracheal intubation, dialysis, vasopressors and inotrops, surgery, antimicrobial therapy, transfusion of blood products, enteral or parenteral nutrition, cardiopulmonary resuscitation, and IV fluids.

Moment of making decision of WH/WD (8-14 h, 14-20 h, night and weekend), the time from admission to ED to making this decision and the time from processing to withhold or withdrawal life-sustaining treatment to death, were noted. Individuals who made the decision to WH/WD were identified; whether a single emergency physician, a medical staff, and involvement of nursing staff in the decision. Involvement of patients and families in the decision-making process, and the presence of a written account of the decision in the patient's medical record were also noted. The study protocol was approved by the Rabat Morrocan University's Ethics Committee. Informed consent was not required since any intervention or treatment were given to the patients as part of this observational study, and the process of the study did not affect therapeutic decisions.

### Statistical analysis

Data are presented as mean ± standard deviation for variables with a normal distribution, and as median and interquartile range (IQR) for variables with skewed distributions. Parametric or nonparametric tests were used for continuous variables as appropriate after the normality of the distribution was tested by the Kolmogorov-Smirnov test with Lilliefors correction. Statistical differences between groups were evaluated by the chi-square tests for categorical variables. Comparison of group differences for continuous variables was carried out by Student test or the Mann_Whitney U test. Variables with *P *value lower than 0.2 in the univariate analysis were tested in the multivariate analysis. Multivariate analysis was performed using stepwise logistic regression models. A two-tailed *P *value < 0.05 was considered statistically significant. Statistical analyses were carried out using SPSS for Windows (SPSS, Inc., Chicago, IL, USA).

## Results

### Characteristics of patients who died in the ED

During the study period, among the 24 500 patients who were admitted to the ED, 14480 (59.1%) were discharged home, 9758 (39.8) were transferred to other medical or surgical care units, and 85 (0.3%) were excluded. Analysis was therefore conducted on the remaining 177 patients. The mean age of the 177 patients who died on stretchers in the ED was 47 years (ranging from 16 to 83 years) with 100 males (56.5%), and 77 females (43.5%). Table 1 shows the characteristics of these patients. The median APACHE II score was 17 ± 7.5 at admission, and 44.6% of the patients who died in the ED had chronic underlying disease. The most frequent presenting acute medical disorders were, cardiovascular (27.7%); infectious (17%), neurological (14.1%), and traumatic (14.1%).

**Table 1:** Patient characteristics according to whether therapy was limited or not (n = 177)

Characteristics	All (n = 177)	WH/WD (n = 54)	WH (n = 43)	WD (n = 11)
Age, years (mean ± SD)	47 ± 17.5	57.7 ± 2.31	58 ± 17	56.9 ± 5
Gender, n (%)				
Male	100(56.5)	30(55.6)	24(55.8)	6(54.5)
Female	77(43.5)	24(44.4)	19(44.2)	5(45.5)
Prior health condition: CCI				
0	89(50.3)	13(24.1)	12(27.9)	1(9.1)
1	45(25.4)	12(22.2)	10(23.3)	2(18.2)
> 2	43(24.3)	29(53.7)	21(48.8)	8(72.7)
APACHE II (mean ± SD)	17 ± 7.5	20.3 ± 1	21 ± 7.6	17.5 ± 5
Acute medical disorders, n (%)				
Cardiac	49(27.7)	10(18.5)	9(20.9)	1(9.1)
Respiratory	15(8.5)	6(11.1)	3(7)	3(27.3)
Neurological	25(14.1)	17(31.5)	15(34.9)	2(18.2)
Infectious	30(16.9)	6(11.1)	5(11.6)	1(9.1)
Metabolic	15(8.5)	3(5.6)	2(4.7)	1(1.9)
Digestive	18(10.2)	8(14.8)	5(11.6)	3(27.3)
Traumatic	25(14.1)	4(7.4)	4(9.3)	0
Chronic underlying diseases, n (%)				
Heart failure	35(19.7)	21(38.9)	17(39.5)	4(36.1)
Chronic respiratory disease	21(11.9)	4(7.4)	3(7)	1(9.1)
Malignancy	14(7.9)	10(18.5)	7(16.3)	3(27.3)
Liver disease	9(5.1)	5(9.2)	2(4.7)	3(27.3)
Time interval from ED admission to death (hours) median [IQR]	24[8-48]	24[12-48]	12[4-34]	12[4-29]
Time interval from ED admission to the decision WH/WD (hours) median [IQR]	----	8[3-24]	7[2-24]	12[6-24]
Time interval from decision WH/WD to death (hours) median [IQR]	----	12[4-34]	24[12-48]	12[12-76]
Documentation of decision WH/WD in medical record, n (%)	----	1(1.85)	1(5.3)	0
Initiation of the decision-making process WH/WD, n (%)				
Single ED physician	----	23(42.6)	19(44.2)	4(36.4)
Single ED physician with Nursing staff Single ED physician without Nursing staff	---- ----	18 (78.3) 5 (21.7)	14(73.7) 5(26.3)	4(100) 0
Medical staff	----	31(57.4)	24(55.8)	7(63.6)
Involvement in the decision-making process WH/WD*, n (%)				
Nursing staff	----	48(88.9)	37(86)	11(100)
Patient	----	6(11.1)	5(11.7)	1(9.1)
Families	----	38(70.4)	29(67.5)	9(81.8)
Moment of decision WH/WD making, n (%)				
From 8 h to 14 h	----	24(44.4)	19(44.2)	5(45.5)
From 14 h to20 h	----	14(26)	11(25.6)	3(27.3)
Night and weekend	----	16(29.6)	13(30.2)	3(27.3)

CCI: Charlson Index of Comorbidities; APACHE II: Acute Physiology and Chronic Health Evaluation; WH: withholding; WD: withdrawal of life-sustaining treatment. *Several individuals can be involved in the decision making process WH/WD for the same patient.

### Characteristics of patients with WH/WD decisions

**Figure 1:**
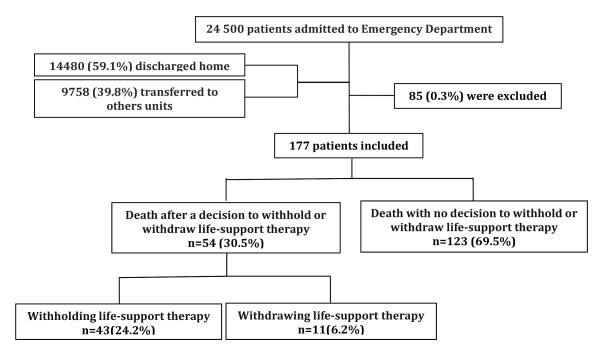
**Trial profile of 24,500 patients admitted to emergency departments during study period**.

A decision to withhold or withdraw life support was taken for 54 patients (30.5%), thus 123 patients died without level-of-care limitation. Withholding concerned 43 patients (24.2%), and withdrawal concerned 11 patients (6.2%) (Figure 1).

Patients who died as a result of withholding and withdrawal of life-sustaining treatment had a median age of 57.7 ± 17 years, of whom 30 (55.5%) were men. The median APACHE II score at admission was 20.3 ± 7.2. The most common chronic underlying diseases were heart failure (14.1%), and malignancy (27.7%), and the most common reasons for admission to the ED among these patients were neurological (14.1%), and cardiovascular diseases (27.7%). Median (IQR) time interval between ED admission and a decision to withhold or to withdraw life-support therapies were respectively of 7 h (IQR: 2-24 h), and 12 h (IQR: 6-24 h). Median (IQR) time interval between a decision to withhold or to withdraw life-support therapies and death were respectively of 24 h (IQR: 12-48 h), and 12 h (IQR: 12-76 h). Criteria used to justify limiting life-support therapies for patients who died in ED were reported in table 2. The decision to limit life-support procedures was recorded in the medical file for only one patient. Life-sustaining treatment modalities withheld or withdrawn are shown in Table 3. The most common modalities withheld or withdrawn life-support therapy were mechanical ventilation in 30 cases (17%), vasopressor and inotrops infusion in 28 cases (15.8%).

**Table 2:** Criteria used to justify limiting life-support therapies for patients who died in ED

Criteria*	N (%)
Principal acute presenting medical disorder	0
Expected irreversibility of acute disorder in the first 24 h	23(42.6)
Age	16(29.6)
Previous functional limitation	3(5.6)
Underlying chronic disease	17(31.5)
Absence of improvement following a period of active treatment	33(61.1)
Underlying disease expected to be fatal in the following 6 months	0
Recovery but expected quality of life unacceptably poor	18(33.3)
Level of care considered to be maximal (more aggressive therapy would be unreasonable)	3(5.6)
High cost of care	8(14.8)

*Several criteria can be used for the same patient to justify the decision making process WH/WD; ED: emergency departments.

**Table 3:** Life support modalities withheld or withdrawn for patients who died in ED

Modalities*	WH	WD
Endotracheal intubation	0	0
Mechanical ventilation	29(16.4)	1(0.6)
Antimicrobial therapy	4(2.3)	8(4.5)
Intravenous fluid expansion	1(0.6)	0
Transfusion of blood products	4(2.3)	3(1.7)
Vasopressor and inotrops infusion	15(8.5)	13(7.3)
Cardiopulmonary resuscitation	2(4.6)	0
Renal replacement therapy	3(1.7)	1(0.6)
Nutrition (enteral or parenteral)	0	1(0.6)
Surgery	14(8.4)	0

*Several Life support modalities withheld or withdrawn can be used for the same patient.

WH: withholding; WD: withdrawal of life-sustaining treatment; ED: emergency departments.

The decision to withhold and withdraw life-sustaining treatment was made by a single physician in 23 cases (42.6%), by medical staff in 31 cases (57.4%). The nursing staff was involved in these decisions in 48 cases (88.9%). Involvement of patients and families in the decision making process are presented in Table 1. Six patients (11.1%) participated in treatment decisions. In 16 cases (29.6%) the family was not involved, and the decision to withhold or withdraw life-sustaining treatment rested on the emergency medical staff and the primary physician.

The reasons for making a decision to withhold or withdraw life support were absence of improvement following a period of active treatment in 33 cases (61.1%), and expected irreversibility of acute disorder in the first 24 h in 23 cases (42.6%) (Table 3). On average, the physicians have chosen 2.5 ± 1.25 (range 1-6) criteria to justify their decisions to withhold or withdraw life-sustaining treatments (Table 3).

Patients in whom therapy was limited had a statistically significantly older age (P < 0.001), a higher CCI (P < 0.001), and a higher APACHE II score at admission (P < 0.001), had a malignancy and a cardiovascular chronic underlying diseases, and were more likely to be admitted with a neurological acute medical diseases (P < 0.001). Patients who received full support were more likely to be admitted with either a cardiovascular, infectious or trauma diagnosis. Table 4 lists the demographic and clinical characteristics of patients according to whether therapy was limited or not.

**Table 4:** The factors associated with withholding and/or withdrawing decisions performed on 177 patients who died in ED in univariate analysis

Characteristics	WH/WD (n = 54)	No WH/WD (n = 123)	*P*
Age, years (mean ± SD)	57.7 ± 17	42.5 ± 15.6	< 0.001
Gender			
Male	30(55.5)	70(5.7)	0.8
Female	24(44.4)	53(43.1)	
Prior health condition: CCI			< 0.001
0	13 (24.1)	76(61.8)	
1	12 (22.2)	33(26.8)	
> 2	29 (53.7)	14(11.4)	
APACHE II	20.3 ± 7.2	16 ± 7.2	< 0.001
Acute medical disorders			0.028
Cardiac	10(18.5)	39(31.7)	
Respiratory	6(11.1)	9(7.3)	
Neurological	17(31.4)	8(6.5)	
Infectious	6(11.1)	24(19.5)	
Metabolic	3(5.5)	12(9.7)	
Digestive	8(14.8)	10(8.2)	
Traumatic	4(7.4)	21(17.1)	
Chronic underlying diseases			< 0.001
Heart failure	21(38.9)	14(11.4)	
Chronic respiratory disease	4(7.4)	17(13.8)	
Malignancy	10(18.5)	4(3.3)	
Liver disease	5(9.3)	4(3.2)	
Time interval from ED admission to death	24[12-48]	24[6-48]	0.078

CCI: Charlson Comorbidities Index; APACHE II: Acute Physiology and Chronic Health Evaluation;

ED: emergency departments.

Multivariate logistic regression for individual factors associated with WH/WD therapy decisions were older age (OR = 1.1; 95%IC = 1.01-1.07; *P *= 0.001), neurological acute medical disorders (OR = 4.1; 95%IC = 1.48-11.68; P = 0.007), malignancy (OR = 7.7; 95%IC = 1.38-8.54; *P *= 0.002) and cardiovascular chronic underlying diseases (OR = 3.4; 95%IC = 2.06-28.5; *P *= 0.008). Table 5 presents the multivariate logistic regression results.

**Table 5:** The multivariate logistic regression model for the composite outcome of withholding and/or withdrawing decisions performed on 177 patients who died in ED

Characteristics	OR	95% CI	*P *value
Age	1.1	1.01-1.07	0.001
Acute medical disorders			
Neurological*	4.1	1.48-11.68	0.007
Chronic underlying diseases			
Heart failure**	7.7	1.38-8.54	0.002
Malignancy**	3.4	2.06-28.55	0.008

OR: Odds Ratio; 95% CI: 95% Confidence Interval; * reference category for acute medical disorders: metabolic disorders;

** Reference category for Chronic underlying diseases: Chronic respiratory disease; ED: emergency departments.

## Discussion

This article reports the results of the first Moroccan observational study concerning the decision of withholding and withdrawal life-sustaining treatment in an Emergency Department. Many ICU studies have focused on decisions to limit life-support treatments in Western countries [11-19,22], and Arabic countries [25,27,31]. However, few studies have focused on WH/WD decisions in the ED in Western countries [4-6,8,23,24,32-34], and to our knowledge, no clinical studies in ED have been reported from Arabic countries.

The main finding of this study was that 30.5% of the ED deaths were preceded by a decision to withhold or withdraw life-support therapies; this frequency is lower than reported by Le Conte et al (78.8%) [7]. Patients who died following these decisions were elderly, with malignancy and cardiac chronic underlying diseases, and neurological acute medical disorders. Clinical factors associated with such a decision are consistent with previous published studies in foreign countries [8,10]. Predicting individual outcomes from critical illnesses remains an imprecise science, but an EOL decision can more easily be justified when the physician concludes that the patient is unresponsive to treatment or has severe neurological injury [22].

Morocco is an Arab Muslim country where religious and cultural issues often play a vital role in decision making by families and physicians [27]. Islamic bioethics is an extension of Shariah (Islamic law), which is itself based on:

(1) The Quran: the Holy Text believed by Muslims to be the direct word of God.

(2) The Sunnah: the aspects of Islamic law based on the Prophet Muhammad's words or acts.

(3) The Ijtihad: the law of deductive logic [35]. In this, learned scholars or *Ulema *are charged with interpreting and disseminating religious teachings. The resolution of bioethical issues, is left to qualified scholars of religious law, who are called upon to provide rulings on whether a proposed action is forbidden, discouraged, neutral, recommended or obligatory [36].

Islamic bioethics emphasizes the importance of preventing illness, but when prevention fails, it provides guidance not only to the practising physician but also to the patient. The physician understands the duty to strive to heal, acknowledging God as the ultimate healer [36]. In 1987 a US based Muslim thinker expressed the view that unnecessary artificial prolongation of life is not in keeping with the spirit of Islam, unless there is evidence that a reasonable quality of life will result [37]. Islamic law permits withdrawal of futile and disproportionate treatment on the basis of the consent of the immediate family members who act on the professional advice of the physician in charge of the case [38].

The figure of nursing involvement in 89% of the cases was surprisingly high, previous studies from Europe have much lower figures [8,22,32]. This high rates, could be related to the relatively young age of our emergency doctors (mean of age: 32 years), who benefits from the nurse experience 50 years on average. Generally, the ED staff did not feel prepared for caring for the dying in the ED. Nursing staff relied on learning from others and experience [23]. Many US papers have recommended participation of the nursing staff in ethical decisions [21,39,40].

We observed obvious ethical limitations in the life-sustaining treatment decision-making processes. First, a substantial portion 21.7% of decisions to limit care was taken by a single physician, with no consultation with the medical or nursing staff. A second worrying finding of this study was that 29.6% of the decisions were taken during night and weekend, which suggests at least some degree of haste. Third, an ethical process entails information and consent of patients, families, or both. In our study only six patients were consulted, and relatives were included in EOL decision in 70% of cases, as reported in others ED studies. However, in our study the reasons for non-participation of patients and families have not been recorded. EOL care requires a great deal of collaboration and communication between the patient, his or her family, and other parties, which becomes extremely difficult in the emergency department given the time constraints [24]. Some patients have seen many doctors and specialists, but no one person can provide the whole picture and help with the decision-making process. Such cases are challenging and time-consuming and require many decisions to be made in a hectic ED environment [24]. The absence of Moroccan guidelines governing the relationship between physician, patient, and family, can explains the low participation of the patient and his family in the decision. Whether or not a doctor can prolong life by introducing aggressive invasive treatments without causing further harm is a joint decision made by all associated with the patient. In some instances the matter is even referred to the religious leaders, who provide prescriptive rulings for the families' consideration [38]. These judgments demand that decision-makers balance important ethical and legal principles such as the sanctity of life, the right of a patient to determine how he/she shall be treated, and the expectation that a doctor's first consideration will be the welfare or best interests of the patient [36]. Fourth, only one decision was notified in the medical record, which may reflect the reluctance of physicians to record their decisions in the Moroccan legal circumstances.

The similarities between our results and those in western countries suggest similarities between Islamic physicians and other Western physicians in EOL decisions. Although Islam has some doctrinal differences from Judaism and Christianity. The 3 monotheistic religions, Judaism, Christianity and Islam, believe in the same God and shares essentially the same code of morality [36].

The finding from our study that WH/WD decisions are done in emergency clinical practice, whatever their frequency, is striking.

In our study, we found that withholding (24.2%) was making rather than withdrawing treatment decision (6.2%). This distinction between withholding and withdrawing treatment was also reported from previous studies [12,18,20,21,31], and could be explained by difficulties encountered by emergency physicians. ED are dedicated to making rapid decisions in a high-stress, fast paced environment and for caring for unexpected illnesses or injuries. However, Physicians often lack crucial data concerning the patient's earlier state of health and autonomy.

## Limitations

Our method to evaluate WH/WD in ED presented some limitations. First, it was a single-centre study. Second, the staff at the ED was not aware of this study at the initiation. But since we did interview the MD's after each death, this could possibly have influenced the answers during the study, since those interviewed at the end of the study now knew which questions they were asked. This may be one of the reasons for the high proportion of nurse involvement, since the MD's knew they would be asked this question. Third, it was a limited number of charts to analyze. This pilot study will be followed by a multicenter study including several Moroccan ED. Data collected from this study will reflect more accurately the practice of all ED physicians regarding withholding and withdrawal of life-sustaining treatment. Previous studies demonstrated high variability in end-of-life care between various groups of physicians in the same country [14,20]. Fourth, the reasons for non-participation of patients and their families have not been recorded. Finally, this study did not investigate all aspects of WH/WD treatment practices. Further studies should focus on specific issues such as the impact of oriental social values and religious Muslim beliefs on the involvement of family members and on refusal of withdrawal life-sustaining treatment.

## Conclusions

Religious beliefs and the lack of guidelines and official Moroccan laws could explain the ethical limitations of the decision-making process recorded in this study. WH/WD decisions are difficult to implement in the ED owing to the absence of an ongoing long-term relationship with the patient and lack of time, but are undeniably an integrated part of medical activity. Many Muslim patients may not be aware of contemporary rulings on bioethical issues. If the community has religious leaders or its own social workers, these can be useful sources. Hospitals should keep their contact numbers close at hand, especially in emergency departments [36]. When withholding or withdrawal of life-sustaining treatment is indicated, coupled with the associated ethical issues and emotional burden for the families, this emphasizes the need to continuously evaluate the implementation and process of withholding and withdrawal of life-sustaining treatment in emergency medical practice. The conditions of life-sustaining treatment must be governed and explained by the Moroccan law; an unified procedure must be established by introduction of scientific guidelines and recommendations adapted to ED setting. Studies of physicians' attitudes and the perceptions of patients and families are necessary to elaborate guidelines, and to clarify the legal position about end-of-life decisions in ED.

## Abbreviations

ED: Emergency department; APACHE II: Acute Physiology and Chronic Health Evaluation II; CCI: Charlson Comorbidities Index; WH: withholding; WD: withdrawing; IV: intravenous; h: hours; IQR: interquartile range; OR: odds ratio; CI: confidence Interval; ICU: intensive care unit; EOL: end of life.

## Competing interests

The authors declare that they have no competing interests.

## Authors' contributions

ND and JB contributed equally to the work. ND participated in the design of the study, and acquisition of data. JB draft the manuscript. BA participated in the acquisition of data. TD, KA, NM, and AZ participated in the coordination of data. ABB participated in the coordination of the study. AAZ participated in the design of the study, and performed the statistical analysis. RA conceived of the study, participated in the design of the study, performed the statistical analysis and interpretation of data, and gave the final approval of the manuscript. All authors read and approved the final manuscript

## Pre-publication history

The pre-publication history for this paper can be accessed here:

http://www.biomedcentral.com/1471-227X/11/12/prepub
